# Epitope Mapping of *Senecavirus A* 3A Protein Using Monoclonal Antibodies

**DOI:** 10.1155/tbed/3398924

**Published:** 2025-05-07

**Authors:** Liang Meng, Xiao-Xiao Tian, Xu-Yan Xiang, Xin-Yu Qi, Han-Rong Zhou, Pei-Yu Xiao, Tong-Qing An, Fan-Dan Meng, Hai-Wei Wang

**Affiliations:** ^1^State Key Laboratory for Animal Disease Control and Prevention, Harbin Veterinary Research Institute, The Chinese Academy of Agricultural Sciences, Harbin 150069, China; ^2^Heilongjiang Provincial Key Laboratory of Veterinary Immunology, Harbin 150069, China

**Keywords:** 3A protein, epitope, monoclonal antibody, *Senecavirus A*

## Abstract

*Senecavirus A* (SVA), an emerging pathogen causing vesicular disease in pigs, poses a significant threat to the swine industry. The nonstructural protein 3A of SVA plays an essential role in the viral replication cycle. In this study, we immunized mice with the prepared SVA 3A protein and produced two monoclonal antibodies (mAbs), AG4 and 2F3. MAb AG4 showed specific reactivity to the linear and conformational 3A protein, whereas mAb 2F3 did not recognize linear epitope of 3A protein. Through truncated 3A protein expression and alanine mutation analysis, we identified ^1^SPNEND^6^ as the minimal motif recognized by mAb AG4, with Asn^3^ being the critical residue. Additionally, we demonstrated that mAb 2F3 failed to recognize the SVA mutant with the ^75^QEETEG^80^ deletion in 3A protein, indicating that ^75^QEETEG^80^ constitutes an essential epitope for mAb 2F3. Further deletion analysis confirmed that ^75^QE^76^ is the crucial motif for mAb 2F3 recognition. Moreover, we found that ^1^SPNEND^6^ and ^75^QEETEG^80^ are highly conserved among different SVA strains and are exposed on the surface of the 3A protein. This study contributes to further explore the function of SVA 3A protein and develop diagnostic tools for SVA detection.

**Summary**

• Since its discovery in 2002, *Senecavirus A* (SVA) has disseminated extensively across numerous countries. Despite its widespread occurrence, no commercial vaccines are currently available against SVA.
• The 3A protein, known to elicit robust and long-lasting antibody responses in vivo, has been identified as an ideal diagnostic target. Additionally, 3A plays a crucial role in the viral replication cycle.
• We obtained two monoclonal antibodies (mAbs) against the 3A protein and identified the B cell epitopes.
• These findings can help us understand the 3A protein's function in viral replication and develop diagnostic tools.

## 1. Introduction

*Senecavirus A* (SVA), also known as *Seneca Valley virus* (SVV), is the sole member of the genus *Senecavirus* within the family Picornaviridae [1]. SVA infection in swine causes vesicular lesions and ulcerations, which clinically indistinguishable from other vesicular diseases, including foot-and-mouth disease (FMD) and vesicular exanthema of swine (VES), thereby complicating diagnosis [2–4]. Since the initial report of an SVA epidemic in Canada in 2007, the SVA has spread rapidly to the United States, Brazil, Thailand, Vietnam, China, and several other countries [5–10], posing a significant risk to the global swine industry.

The genome of SVA is characterized by a single-stranded, positive-sense RNA ~7200 nucleotides in length, comprising a 5′ untranslated region (5′ UTR), an open reading frame (ORF) encoding a polyprecursor protein, and a 3′ untranslated region (3′ UTR). The ORF includes a leader protein (L) and three polyproteins, including P1, P2, and P3. The P1 polyprotein is processed by the 3C protease into VP0, VP3, and VP1, with VP0 subsequently cleaved into VP4 and VP2. The P2 and P3 polyproteins are proteolytically cleaved to produce seven nonstructural proteins: 2A, 2B, 2C, 3A, 3B, 3C, and 3D [1, 11]. Among these, the 3A protein is crucial for viral replication. Preliminary research suggests that 3A facilitates immune evasion and enhances viral replication by degrading G3BP1 through modulation of the autophagy-associated protein LRRC25 [12].

Current research on SVA monoclonal antibodies (mAbs) has been predominantly focused on structural proteins (VP2, VP3, and VP1) [13–15], whereas nonstructural proteins, which do not directly contribute to viral particle formation, have received comparatively less investigative attention. Notably, the 3A protein warrants special consideration due to its critical role in the viral life cycle and potential diagnostic applications [16]. Nonstructural proteins are abundantly expressed during early infection stages, positioning 3A-specific mAbs as valuable tools for early pathogen detection [17]. Furthermore, the development of 3A mAbs provides an essential tool for elucidating the role of 3A protein in the SVA life cycle and for developing detection methods. Therefore, this study aims to generate mAbs specific to the SVA 3A protein and finely map its B cell epitopes, thereby laying a foundation for further investigation the function of 3A protein.

## 2. Materials and Methods

### 2.1. Virus and Cells

The wild-type SVA/HLJ/CHA/2016 (GenBank accession number: KY419132) [18] and SVA VP2 mAb 2C7 [13] were kept in our lab as described previously. The BHK-21 and SP2/0 cells were maintained in Dulbecco's Modified Eagle Medium (DMEM) supplemented with 10% fetal calf serum (Gibco-BRL, Grand Island, NY, USA).

### 2.2. Cloning, Expression, and Purification of 3A Protein

Based on the SVA/HLJ/CHA/2016 strain, forward primer sequences (5′- CAGCAAATGGGTCGCGGATCCGAATTCAGCCCTAACGAGAACGACGGCAC-3′) and reverse primer (5′-CAGTGGTGGTGGTGGTGGTGCTCGAGCTC GCTCCTAGGCGCTTTAGCAGGCTCCTC-3′) were designed to amplify the gene encoding the 3A protein via polymerase chain reaction (PCR). The amplified 3A fragment was cloned into the pET24a (+) expression vector using *BamH* I and *Xho* I restriction sites, to construct the recombinant prokaryotic expression plasmid pET24-His-3A. The recombinant plasmid was transformed into the *E. coli* BL21 (DE3). Monoclonal colonies were inoculated into Luria–Bertani (LB) liquid medium and cultured until the optical density at 600 nm (OD600) reached between 0.6 and 0.8. Protein expression was induced by adding isopropyl β-D-1-thiogalactopyranoside (IPTG) to a final concentration of 0.1 mmol/L. After 20 h of induction, cells were harvested by centrifugation at 4°C and 6000 g for 5 min. The cell pellet was resuspended in 10 mL of Buffer A (250 mM NaCl, 50 mM Tris, pH 8.0) and lysed by ultrasonication. The lysate was then centrifuged at 12,000 rpm for 10 min at 4°C to separate soluble and insoluble fractions. The 3A protein was affinity-purified using High Affinity Ni-Charged Resin FF (Genscript, China) and eluted with a gradient of imidazole (Solarbio, China) in Buffer A. The expression and purification of 3A protein were analyzed by sodium dodecyl sulfate–polyacrylamide gel electrophoresis (SDS-PAGE) and Western blot.

### 2.3. MAb Preparation

Six-week-old female BALB/c mice were immunized with 50 µg of the purified-recombinant 3A protein emulsified with an equal volume of adjuvant Montanide ISA-201 (SEPPIC, France) by intramuscular injection. Two boosters of the adjuvant emulsified antigen were administered at 2-week intervals. Two weeks after the third immunization, the mice received an intraperitoneal boost with 100 µg of antigen alone. Three days postboost, spleen cells from the immunized mice were fused with SP2/0 myeloma cells at a 1:5 ratio using PEG6000 (Sigma-Aldrich, USA). The fusion mixture was incubated in DMEM supplemented with 20% fetal bovine serum (FBS), hypoxanthine–aminopterin–thymidine (HAT) (Sigma-Aldrich, USA), and 10% hybridoma feeder (Biodragon, China) at 37°C. Indirect immunofluorescence assay (IFA) was used to screen the hybridomas that specifically produce mAbs against the 3A protein. Positive hybridomas were subjected to three rounds of cloning by limiting dilution to ensure monoclonality.

### 2.4. SDS-PAGE and Western Blot

Approximately equal amounts of each protein sample were subjected to 12% SDS-PAGE). The gel was either stained with Coomassie blue or transferred to PVDF membranes (Millipore, MA, USA). The membrane was blocked with 5% nonfat milk in phosphate-buffered saline (PBS) overnight at 4°C and then incubated with anti-His antibody (Sigma-Aldrich, USA) or hybridoma cells supernatant at 37°C for 1 h. After three washes with PBST, the membrane was probed with a 1:10,000 dilution of DyLight 800-labeled antimouse IgG (H + L) antibody (KPL, USA) at 37°C for 1 h. The reactivity was visualized using the Odyssey CLx Image Studio (LI-COR, USA).

### 2.5. IFA

BHK-21 cells in 96-well plates were infected with SVA at a multiplicity of infection (MOI) of 0.1. The cells were fixed with ice-cold anhydrous ethanol for 15 min at 4°C and air dried. Subsequently, 50 µL/well of hybridoma cell supernatant was added and incubated for 1 h at 37°C. After washing with PBS, 50 µL/well of FITC-conjugated goat antimouse IgG (Sigma, St. Louis, MO, USA) at a 1:200 dilution was added and incubated for 1 h at 37°C. The plates were washed three times with PBS and examined under an EVOS FL Auto 2 Cell Image System.

### 2.6. Epitope Mapping

A series of truncated 3A fragments were generated through primer amplification or oligonucleotide annealing and subsequently cloned into pEGX-6p-1 vector using *EcoR* I and *Not* I restriction sites. All primer sequences are listed in Table S1. The recombinant truncated proteins were expressed in *E. coli* BL21 (DE3). These recombinant truncated proteins were then used to map the epitopes of mAbs by Western blotting.

### 2.7. Construction and Rescue of Epitope-Deleted Viruses

Primers were designed based on the gene sequence of the SVA/HLJ/CHA/2016 strain (Table S2). These primers were used to amplify different epitope deletion fragments, which were cloned into the SVA-I212V/S460L infectious clone backbone through homologous recombination. The recombinant clones were designated pSVA-*Δ*3A(3-8), pSVA-*Δ*3A(75-80), and pSVA*Δ*3A(77-86).

BHK-21 cells in 6-well plates were transfected with the constructed clones using Lipofectamine 3000, following the manufacturer's instructions (Life Technologies, NY, USA). The cells were monitored daily for cytopathic effect (CPE) observation. Epitope deletion viruses were harvested once significant CPE was observed. These rescued viruses were serially passaged 12 times in BHK-21 cells, and the stability was confirmed by sequencing the 3A region.

### 2.8. Replication Dynamics of Epitope Deletion Virus

BHK-21 cells were seeded in 6-well plates until the cells reached 80% confluence and then infected with either wild-type virus or epitope-deleted virus at an MOI of 0.1 to assess viral replication dynamics. After a 1-h incubation at 37°C, the plates were washed three times with PBS and cultured in DMEM with 2% FBS. Samples were harvested at various time points, and viral titers were determined using the 50% tissue culture infective dose (TCID_50_) assay. TCID_50_ values were calculated using the Reed–Muench method.

### 2.9. Viral Plaque Assay

BHK-21 cells were seeded in 6-well tissue culture plates until cell monolayers reached 100% confluency and then infected with 10-fold serial dilutions of SVA-WT and rSVA -*Δ*3A(75-80). After a 1-h incubation at 37°C, the infected cells were washed three times with PBS to remove unbound virus particles and then overlaid with DMEM supplemented with 2% FBS and 5% methylcellulose. When plaques were observed, the DMEM and methylcellulose overlay was carefully removed. The cells were washed thrice with PBS and stained with crystal violet.

### 2.10. Biological Information Analysis of 3A

The secondary structure and epitopes of the SVA 3A protein were predicted and analyzed using the online software suite PSIPRED (http://bioinf.cs.ucl.ac.uk/psipred/) and Bepipred (https://services.healthtech.dtu.dk/services/BepiPred-3.0/). Concurrently, the surface accessibility, hydrophilicity/hydrophobicity, and antigenic index of the 3A protein were evaluated using the DNASTAR Protean software. Homology modeling for the 3A protein was conducted using the I-TASSER server to provide a structural framework for further analysis. Comparative analysis of the 3A amino acid sequences across various SVA isolates was performed using MEGA 7.0 software.

### 2.11. Statistical Analysis

All experiments were set up with three replicates, and each experiment was independently repeated at least three times. Statistical analysis was conducted using two-way analyses of variance with GraphPad Prism 8.0 software. A *p* value of less than 0.05 for each test was considered statistically significant, and significant differences between groups are indicated by *⁣*^*∗∗∗*^*p* < 0.001, *⁣*^*∗∗*^*p* < 0.01, and *⁣*^*∗*^*p* < 0.05.

## 3. Results

### 3.1. A Protein Expression and Purification

**Figure 1:**
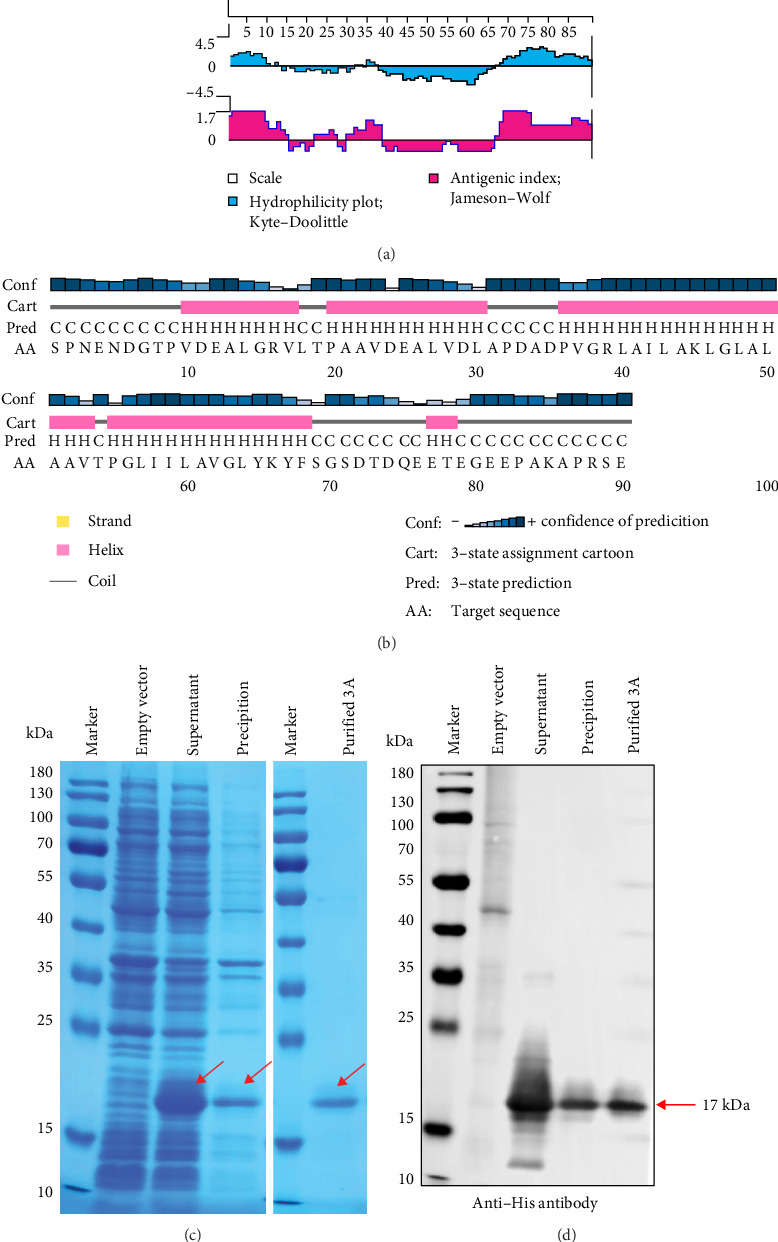
Characterization, expression, and purification of SVA 3A protein. (A) Analysis of antigenicity and hydrophilicity of 3A protein using Protean software. (B) The secondary structure of the 3A protein was analyzed by PSIPRED online software. (C, D) The expression in *E. coli* and purification of 3A protein were analyzed by SDS-PAGE (C) and Western blot (D).

In general, the antigenicity of a protein is mainly determined by the hydrophilicity, surface accessibility, and flexibility of its surface amino acid residues. Hydrophilic regions and random coil regions are more likely to be epitopes due to their high surface accessibility and flexibility [19]. We systematically evaluated the immunogenic potential of 3A protein by comprehensively analyzing its antigenic index, hydrophilicity, and secondary structure composition. Prediction analysis showed that the 3A protein consists of 59% alpha-helix region and 41% random coils (Figure 1B) and that the amino and carboxyl termini of the 3A protein exhibited high antigenicity and hydrophilicity (Figure 1A). The results showed that the 3A protein had good immunogenicity, and the epitopes were mainly concentrated in the amino and carboxyl termini.

We expressed 3A recombinant protein with 6His tag in *E. coli* and purified by nickel resin affinity chromatography. Expression and purification of the 3A protein were assessed using SDS-PAGE and Western blot (Figure 1C,D). Results showed that the recombinant 3A protein had an approximate molecular weight of 17 kDa. Notably, the protein was predominantly soluble, and the purified 3A protein showed high purity.

### 3.2. Generation of MAbs Against 3A Protein

**Figure 2:**
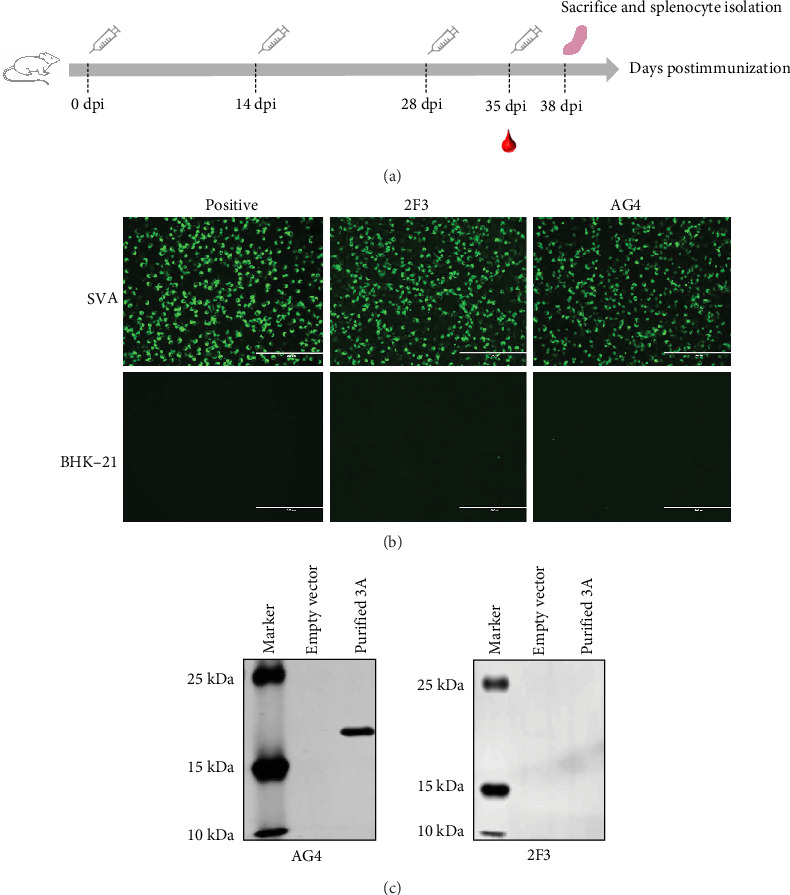
Preparation and functional analysis of mAbs. (A) Immunization protocol for mice. (B) BHK-21 cells were infected with SVA-WT at an MOI of 0.1. Cells were harvested and fixed at 24 hpi for IFA analysis using mAbs AG4 and 2F3 as primary antibody. (C) Western blot analysis of the reactivity of mAbs AG4 and 2F3 with the linear 3A protein.

The mice immunization strategy is shown in Figure 2A. Hybridoma cell supernatants were screened for specific antibodies using an IFA. Two hybridoma cell clones, AG4 and 2F3, secreted antibodies specifically recognized the SVA. After three rounds of subclone screening and multiple subclone passages, the ability of the hybridoma cells AG4 and 2F3 to secrete antibodies was maintained. The specificity of the mAbs was further validated using IFA and Western blot. IFA results showed that both mAb AG4 and 2F3 only reacted with SVA-infected BHK-21 cells instead of the uninfected cells (Figure 2B), indicating the high specificity of the mAbs. Additionally, Western blot analysis confirmed that mAb AG4 specifically reacted with the linear 3A recombinant protein, but mAb 2F3 could not (Figure 2C), which indicates that mAb 2F3 may recognize the conformational epitope on 3A protein. We also assessed the virus-neutralizing capacity of mAbs AG4 and 2F3. However, neither antibody showed neutralizing activity at a 1:2 dilution (against 50 TCID_50_) (data not shown).

### 3.3. Identification of Epitopes Recognized by MAb AG4

**Figure 3:**
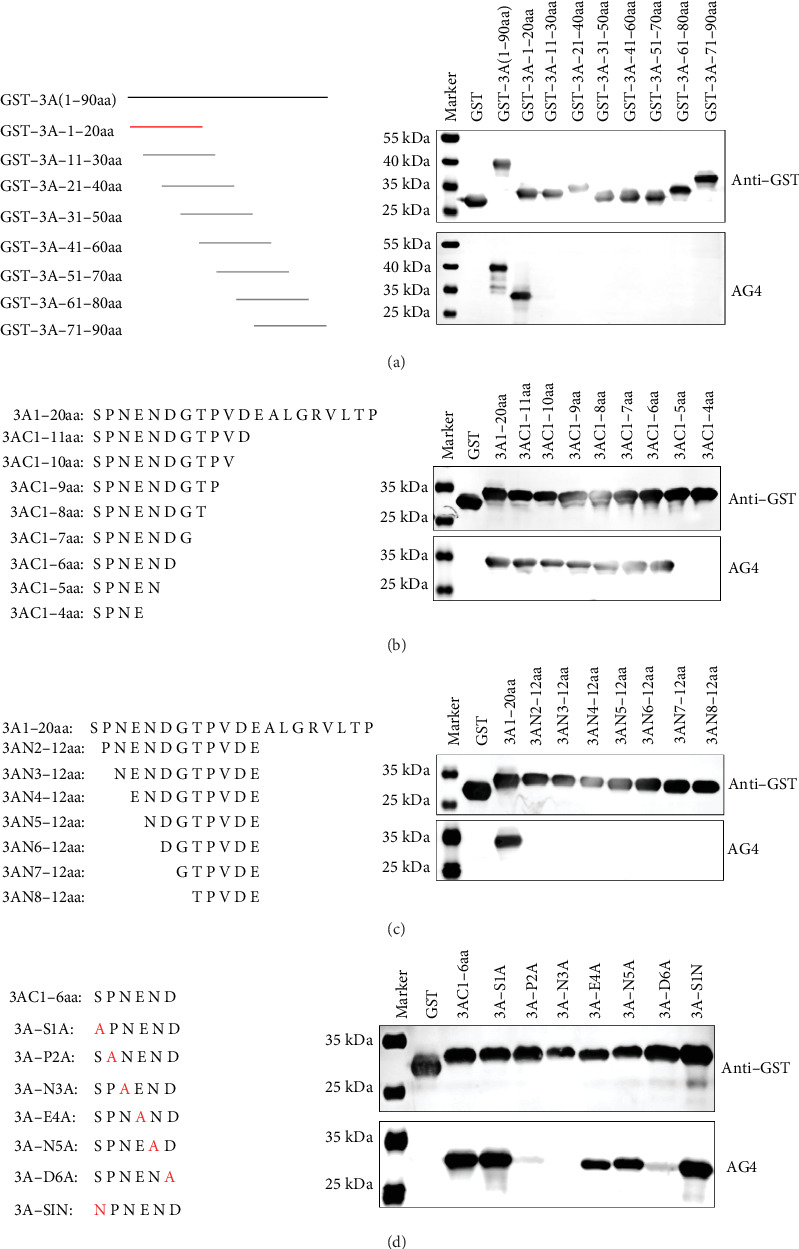
Epitope mapping of mAb AG4. (A) Eight overlapping truncated fragments of the 3A protein were expressed in *E. coli* BL21 and analyzed by Western blot using mAb AG4. (B, C) Identification of the minimal epitope recognized by mAb AG4. The 3A1-20aa fragment was sequentially truncated from the C-terminus (B) and N-terminus (C). (D) Alanine scanning was performed to identify the critical amino acids recognized by mAb AG4, as well as to mutate residue ^1^S to ^1^N.

To map the epitopes recognized by mAb AG4, eight truncated 3A proteins fused with GST were expressed, each with a 10 amino acid overlapping, and subjected to Western blot. MAb AG4 specifically recognized the recombinant protein GST-3A-1-20aa (Figure 3A). Additionally, we performed stepwise single amino acid truncations from the amino- and carboxyl-terminus of the 1–12 aa motif, respectively. Specifically, mAb AG4 recognized the 3AC1-6aa fusion peptide until truncation from the C-terminus to the sixth residue D (Figure 3B,C). However, mAb AG4 lost reactivity with the truncated protein lacking the fifth residue Asn. Similarly, truncation of the first amino acid Ser from the N-terminus also resulted in loss of reactivity with mAb AG4. These results indicated that the minimal epitope recognized by mAb AG4 is ^1^SPNEND^6^.

To identify critical amino acids recognized by mAb AG4, we performed alanine scanning of ^1^SPNEND^6^. Additionally, we mutated residue Ser^1^ to Asn. The results showed that mutating Ser^1^ to Ala or Asn did not affect mAb AG4 recognition of the epitope, whereas mutating Asn^3^ to Ala resulted in loss of mAb AG4 reactivity (Figure 3D), indicating Asn^3^ is critical for the mAb AG4 recognition. Moreover, mutating Pro^2^ and Asp^6^ to Ala significantly reduced mAb AG4 binding activity (Figure 3D), suggesting that Pro^2^ and Asp^6^ are important for mAb AG4 binding.

### 3.4. MAb 2F3 Failed to React With SVA Mutant With 3A Protein C-Terminal Deletion

**Figure 4:**
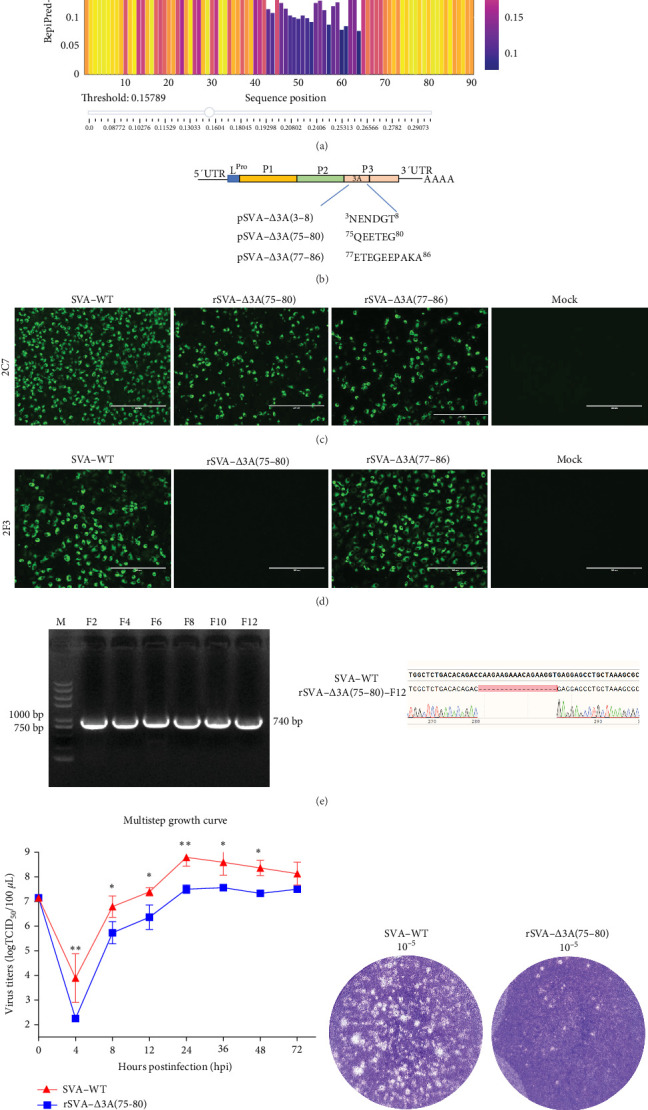
Construction of 3A epitope deletion mutants and epitope mapping of mAb 2F3. (A) The B cell epitope of SVA 3A was predicted using the BepiPred 3.0 tool. (B) Schematic diagram of the construction of 3A epitope deletion infectious clones. (C) The constructed infectious clones were transfected into BHK-21 cells for virus rescue. Only the pSVA-*Δ*3A(75-80) and pSVA-*Δ*3A(77-86) clones successfully rescued the virus, which were named rSVA-*Δ*3A(75-80) and rSVA-*Δ*3A(77-86), respectively. The infection of the rescued virus was detected by mAb 2C7. (D) To identify the epitope recognized by mAb 2F3, BHK-21 cells were infected with the rescued epitope deletion viruses rSVA-*Δ*3A(75-80) and rSVA-*Δ*3A(77-86) at an MOI of 0.1. Cells were harvested and fixed at 12 hpi for IFA using mAb 2F3. (E) The rSVA-*Δ*3A(75-80) virus was serially passaged 12 times in BHK-21 cells, and the genetic stability analysis of the epitope deletion virus rSVA-*Δ*3A(75-80) was conducted. Nucleic acids were extracted and PCR amplified from passages 2, 4, 6, 8, 10, and 12. The PCR product from the 12th passage was sequenced for validation. (F) Growth curve of SVA WT and rSVA-*Δ*3A. BHK-21 cells were infected with SVA-WT and rSVA-*Δ*3A(75-80) at an MOI of 0.01. Samples were collected at various time points postinfection and titrated on BHK-21 cells. The data were presented as the means ± SD (*n* = 3 biological replicates). *p* values were calculated using two-way analyses of variance as *⁣*^*∗*^*p* < 0.05 and *⁣*^*∗∗*^*p* < 0.01. (G) Plaque morphologies of SVA-WT and the epitope deletion mutant rSVA-*Δ*3A(75-80).

Given that mAb 2F3 did not react with the linear 3A recombinant protein (Figure 2C), it is hypothesized that the mAb 2F3 recognized epitope could be conformational. To determine the epitope recognized by 2F3, we first used the online tool Bepipred to predict B cell epitopes of the 3A protein. The results indicated that the 3A epitopes are primarily located at the N-terminus or the C-terminus (Figure 4A). Based on these predictions, several consecutive amino acids with high antigenicity indices from both the N-terminus and C-terminus were selected as potential dominant antigenic epitopes. These regions were then used to construct infectious clones of 3A epitope deletion mutants: pSVA-*Δ*3A(3-8), pSVA-*Δ*3A(75-80), and pSVA-*Δ*3A(77-86) (Figure 4B). The constructed infectious clones were transfected into BHK-21 cells to rescue the SVA 3A-deletion mutants. The results showed that pSVA-*Δ*3A(3-8) was lethal, but pSVA-*Δ*3A(75-80) and pSVA-*Δ*3A(77-86) were successfully rescued and were designated as rSVA-*Δ*3A(75-80) and rSVA-*Δ*3A(77-86), respectively (Figure 4C). IFA using mAb 2F3 as the primary antibody revealed that mAb reacted with the epitope deletion mutant rSVA-*Δ*3A(77-86) but not rSVA-*Δ*3A(75-80) (Figure 4D), indicating that the ^75^QEETEG^80^ deletion is the essential epitope recognized by mAb 2F3. The overlapping sequence between rSVA-*Δ*3A(75-80) and rSVA-*Δ*3A(77-86) is ^77^ETEG^80^; thus, the nonoverlapping motif is ^75^QE^76^. MAb 2F3 failed to react with SVA mutant with ^75^QE^76^ deletion, indicating ^75^QE^76^ is the crucial motif for mAb 2F3 recognition.

Additionally, we assessed the genetic stability and replication dynamics of the deletion mutant rSVA-*Δ*3A(75-80). After 12 serial passages, no additional amino acid deletions, insertions, or mutations were observed (Figure 4E), indicating that pSVA-*Δ*3A(75-80) was genetically stable. Multistep growth curve results showed that the replication dynamics of the deletion mutant were similar to those of SVA-WT, though its replication capacity was approximately one log lower than that of SVA-WT (Figure 4F). Moreover, after infecting BHK-21 cells with the same dilution of virus for 48 h, the number and size of plaques produced by epitope deletion mutant rSVA-*Δ*3A(75-80) were significantly smaller than those produced by SVA-WT (Figure 4G), further demonstrating that the replication ability and growth rate of the deletion mutant were slower compared to SVA-WT.

### 3.5. The Identified Epitopes Are Highly Conserved and Surface-Located in the 3A Proteins

**Figure 5:**
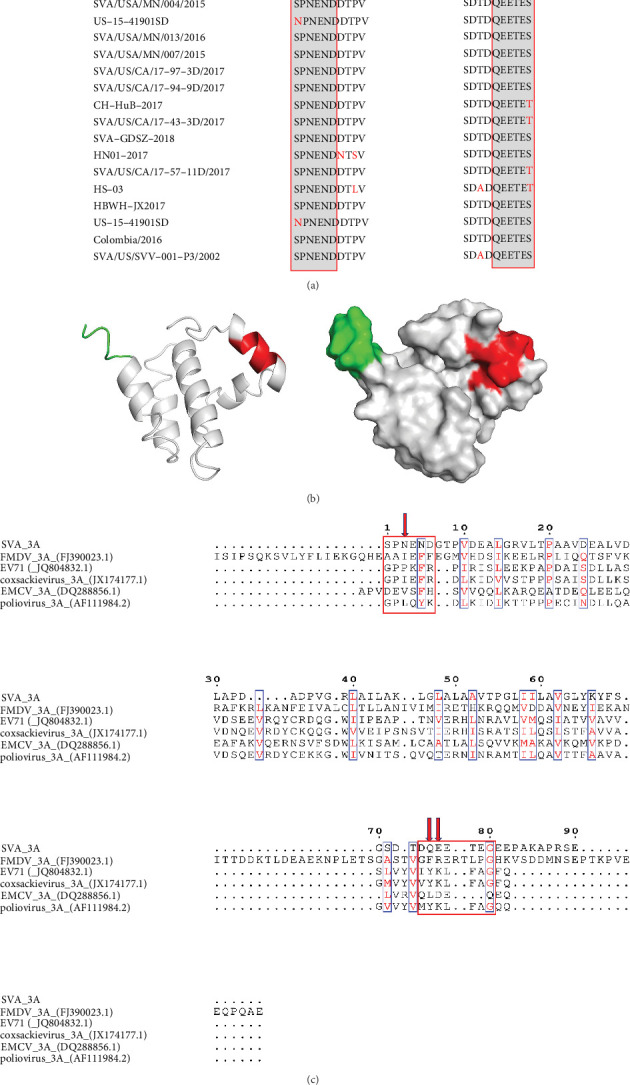
Structural analysis and multiple sequence alignment of SVA 3A. (A) Sequence alignment analysis of the identified epitopes was performed using MEGA 7.0 software. The sequence alignment of the epitope ^1^SPNEND^6^ among different SVA isolates (left) and the alignment of the epitope ^75^QEETEG^80^ among different SVA isolates (right). (B) The detailed positions of the 3A protein epitopes ^1^SPNEND^6^ (red) and ^75^QEETEG^80^ (green) were analyzed, respectively. (C) Sequence alignment analysis of 3A proteins of different picornaviruses. The red arrows indicate the key amino acids identified. The red boxes represent the epitope ^1^SPNEND^6^ and ^75^QEETEG^80^, respectively.

Sequence alignment of the 3A protein from different SVA isolates showed that the epitope ^1^SPNEND^6^ and ^75^QEETEG^80^ are highly conserved, except for a single amino acid substitution (Figure 5A). The 3D structure of the 3A protein was predicted using the I-TASSER server and visualized for analysis. The results revealed that the epitope ^1^SPNEND^6^ is situated in a random coil region, while the epitope ^75^QEETEG^80^ is located within an α-helix. Both epitopes are positioned on the surface of the 3A protein (Figure 5B). Furthermore, we aligned the SVA 3A protein sequence with 3A homologs from representative picornaviruses, including FMDV, enterovirus 71 (EV71), encephalomyocarditis virus (EMCV), poliovirus, and coxsackievirus. The results demonstrated low sequence homology between SVA 3A and other picornaviral 3A proteins. Although the G^80^ residue within the ^75^QEETEG^80^ epitope showed partial overlap in some viruses, the critical residues (Asn^3^ in ^1^SPNEND^6^ and the ^75^QE^76^ motif in ^75^QEETEG^80^) were entirely non-conserved (Figure 5C). This lack of conservation suggests a minimal risk of cross-reactivity.

## 4. Discussion

SVA is affecting the swine industry in many countries and regions around the world, undergoing genetic evolution and viral recombination [20, 21]. Despite extensive research into potential vaccine candidates, no commercial vaccine is currently available, and the lack of differentiating infected from vaccinated animals (DIVA) strategies, complicating disease control and surveillance [22, 23]. FMDV, a member of the *Picornaviridae* family, can tolerate certain amino acid deletions in its 3A protein [24, 25], aiding the development of DIVA marker vaccines [16]. Additionally, the 3AB nonstructural protein, a precursor to 3A, elicits early and sustained antibody responses that are detectable as early as 7-day postinfection [17]. This characteristic of immunogenicity and modifiability make 3A protein an ideal target for early diagnosis and development of DIVA-based SVA vaccine.

In this study, we expressed and purified SVA 3A protein to prepare mAbs, obtaining mAbs AG4 and 2F3. AG4 specifically binds to the linear epitope ^1^SPNEND^6^ on 3A protein, with Asn3 identified as a critical residue for antibody binding. Although an partical overlapping epitope (^5^NDDTPVDEALGR^16^) has been reported [26], our work provides a refined characterization of the AG4-binding epitope, resolving its precise boundaries (^1^SPNEND^6^) and pinpointing Asn^3^ as a key recognition site. This difference in comparison to the previously described regions indicates that the epitopes we have identified are different from each other. Furthermore, we identified a novel conformational epitope, ^75^QEETEG^80^, where the ^75^QE^76^ motif is essential for recognition by 2F3. Sequence alignment and structural analysis revealed that both epitopes are highly conserved among different SVA isolates and are exposed on the surface of the 3A protein (Figure 5A,B), highlighting their significance as antigenic sites.

The FMDV 3A protein is crucial for viral virulence and host specificity, with specific deletions restricting replication in bovine cells and attenuating virulence [27, 28]. In many small RNA viruses, the 3A protein forms the viral replication complex, promoting replication [29–31]. The predicted SVA 3A protein (Figure 1A,B) contains an N-terminal domain, a central transmembrane hydrophobic domain, and a C-terminal domain similar to FMDV 3A protein [32, 33]. Although the function of the C-terminal domain in FMDV remains unclear, the deletion of amino acids 75–80 in rSVA-*Δ*3A(75-80) resulted in reduced replication and growth capacity compared to SVA-WT (Figure 4F,G), indicating the C- terminus region of 3A protein influences the replication of SVA. The specific mechanism requires further investigation. Importantly, mAb 2F3 did not react with virus rSVA-*Δ*3A(75-80) (Figure 4D), indicating the potential of ^75^QEETEG^80^ deletion as a specific marker to distinguish between SVA-WT and rSVA-*Δ*3A(75-80).

This study prepared two mAbs against the 3A protein and identified their antigenic epitopes, laying the foundation for further exploration of the 3A protein's function and potential targets for differential diagnostic methods. The rSVA-*Δ*3A(75-80) with deleted epitope does not react with the mAb 2F3, potentially making it a negative marker to distinguish between wild-type virus infection and vaccine immunity. In future studies, we will validate the potential of the epitope-deleted virus rSVA-*Δ*3A(75-80) as a DIVA-based vaccine candidate and analyze vaccine-induced immunity from wild-type virus infection in vivo and further develop differential diagnostic tools.

## Data Availability

The data that support the findings of this study are available from the corresponding author upon reasonable request.

## Supporting Information

Supporting Information Table S1. The sequence of primers. Table S2. The sequence of primers.
