# Supplementary material for: Epitope Mapping of Senecavirus A 3A Protein Using Monoclonal Antibodies
Source: Transbound Emerg Dis. 2025 May 7;2025:3398924. doi: 10.1155/tbed/3398924 (PMC12077972; doi:10.1155/tbed/3398924)
Supplement: Supporting Information — Table S1. The sequence of primers. Table S2. The sequence of primers. [file 3398924.f1.docx]

**SUPPLEMENTARY INFORMATION for**

**Epitope mapping of *Senecavirus* A 3A protein using monoclonal antibodies**

**Liang Meng ^a^*, Xiaoxiao Tian ^a^*, Xuyan Xiang ^a^, Xinyu Qi ^a^, Hanrong Zhou ^a^, Peiyu Xiao ^a^, Tongqing An ^a,b^ , Fandan Meng ^a#^, Haiwei Wang ^a#^**

**Table S1 The sequence of primers**

| **Primer** | **Sequence (5'→3')** |
| --- | --- |
| GST-3A(1-90 aa) | CCTGGGATCCCCGGAATTCAGCCCTAACGAGAACGACGGCAC |
|  | GATCGTCAGTCAGTCACGATGCGGCCGCTCGCTCCTAGGCGCTTTAG |
| GST-3A-1-20 aa | CGTCGACAGGGGTGCCGTCGTTCTCGTTAGGGCTGAATTCCGGGGATCCCAGGGGCCCCTGGAACAG |
|  | GAACGACGGCACCCCTGTCGACGAGGCGTTGGGTAGAGTTCTCACCCCCGAGCGGCCGCATCGTGACTG |
| GST-3A-11-30 aa | GTAGAGTTCTCACCCCCGCTGCGGTCGACGAGGCGCTTGTCGACCTCGAGCGGCCGCATCGTGACTG |
|  | CAGCGGGGGTGAGAACTCTACCCAACGCCTCGTCGAATTCCGGGGATCCCAGGGGCCCCTGGAACAG |
| GST-3A-21-40 aa | GCTTGTCGACCTCGCTCCAGATGCCGACCCGGTTGGCCGCTTGGAGCGGCCGCATCGTGACTG |
|  | CTGGAGCGAGGTCGACAAGCGCCTCGTCGACCGCAGCGAATTCCGGGGATCCCAGGGGC |
| GST-3A-31-50 aa | GATGCCGACCCGGTTGGCCGCTTGGCTATTCTCGCCAAGCTAGGTCTTGCCCTAGAGCGGCCGCATCGTGACTG |
|  | GCGGCCAACCGGGTCGGCATCTGGAGCGAATTCCGGGGATCCCAGGGGCCCCTGGAACAGAAC |
| GST-3A-41-60 aa | CTCGCCAAGCTAGGTCTTGCCCTAGCTGCGGTCACCCCCGGTTTGATAATCTTGGAGCGGCCGCATCGTGACTGA |
|  | CTAGGGCAAGACCTAGCTTGGCGAGAATAGCGAATTCCGGGGATCCCAGGGGCCCCTGGAACAG |
| GST-3A-51-70 aa | CACCCCCGGTTTGATAATCTTGGCAGTGGGACTCTACAAGTACTTCTCTGGCGAGCGGCCGCATCGTGACTG |
|  | CCAAGATTATCAAACCGGGGGTGACCGCAGCGAATTCCGGGGATCCCAGGGGCCCCTGGAACAG |
| GST-3A-61-80 aa | CTACAAGTACTTCTCTGGCTCTGACACAGACCAAGAAGAAACAGAAGGTGAGCGGCCGCATCGTGACTG |
|  | GAGCCAGAGAAGTACTTGTAGAGTCCCACTGCGAATTCCGGGGATCCCAGGGGCCCCTGGAAC |
| GST-3A-71-90 aa | CAAGAAGAAACAGAAGGTGAGGAGCCTGCTAAAGCGCCTAGGAGCGAGGAGCGGCCGCATCGTGACTG |
|  | CTCACCTTCTGTTTCTTCTTGGTCTGTGTCAGAGAATTCCGGGGATCCCAGGGGCCCCTGGAACAG |
| 3AC1-11aa | AATTCAGCCCTAACGAGAACGACGGCACCCCTGTCGACTAATGAGAGC |
|  | GGCCGCTCTCATTAGTCGACAGGGGTGCCGTCGTTCTCGTTAGGGCTG |
| 3AC1-10aa | AATTCAGCCCTAACGAGAACGACGGCACCCCTGTCTAATGAGAGC |
|  | GGCCGCTCTCATTAGACAGGGGTGCCGTCGTTCTCGTTAGGGCTG |
| 3AC1-9aa | AATTCAGCCCTAACGAGAACGACGGCACCCCTTAATGAGAGC |
|  | GGCCGCTCTCATTAAGGGGTGCCGTCGTTCTCGTTAGGGCTG |
| 3AC1-8aa | AATTCAGCCCTAACGAGAACGACGGCACCTAATGAGAGC |
|  | GGCCGCTCTCATTAGGTGCCGTCGTTCTCGTTAGGGCTG |
| 3AC1-7aa | AATTCAGCCCTAACGAGAACGACGGCTAATGAGAGC |
|  | GGCCGCTCTCATTAGCCGTCGTTCTCGTTAGGGCTG |
| 3AC1-6aa | AATTCAGCCCTAACGAGAACGACTAATGAGAGC |
|  | GGCCGCTCTCATTAGTCGTTCTCGTTAGGGCTG |
| 3AC1-5aa | AATTCAGCCCTAACGAGAACTAATGAGAGC |
|  | GGCCGCTCTCATTAGTTCTCGTTAGGGCTG |
| 3AC1-4aa | AATTCAGCCCTAACGAGTAATGAGAGC |
|  | GGCCGCTCTCATTACTCGTTAGGGCTG |
| 3AN2-12aa | AATTCCCTAACGAGAACGACGGCACCCCTGTCGACGAGTAATGAGAGC |
|  | GGCCGCTCTCATTACTCGTCGACAGGGGTGCCGTCGTTCTCGTTAGGG |
| 3AN3-12aa | AATTCAACGAGAACGACGGCACCCCTGTCGACGAGTAATGAGAGC |
|  | GGCCGCTCTCATTACTCGTCGACAGGGGTGCCGTCGTTCTCGTTG |
| 3AN4-12aa | AATTCGAGAACGACGGCACCCCTGTCGACGAGTAATGAGAGC |
|  | GGCCGCTCTCATTACTCGTCGACAGGGGTGCCGTCGTTCTCG |
| 3AN5-12aa | AATTCAACGACGGCACCCCTGTCGACGAGTAATGAGAGC |
|  | GGCCGCTCTCATTACTCGTCGACAGGGGTGCCGTCGTTG |
| 3AN6-12aa | AATTCGACGGCACCCCTGTCGACGAGTAATGAGAGC |
|  | GGCCGCTCTCATTACTCGTCGACAGGGGTGCCGTCG |
| 3AN7-12aa | AATTCGGCACCCCTGTCGACGAGTAATGAGAGC |
|  | GGCCGCTCTCATTACTCGTCGACAGGGGTGCCG |
| 3AN8-12aa | AATTCACCCCTGTCGACGAGTAATGAGAGC |
|  | GGCCGCTCTCATTACTCGTCGACAGGGGTG |
|  |  |

**Table S2 The sequence of primers**

| **Primer** | **Sequence (5'→3')** |  |
| --- | --- | --- |
| pSVA-△3A(3-8)-F | CTACAGAGCCCTCCTGTCGACGAGGCGTTGGGTAG |  |
| pSVA-△3A(3-8)-R | CTCGTCGACAGGAGGGCTCTGTAGAACCAGAGTCTG |  |
| pSVA-△3A(75-80)-F | CTCTGACACAGACGAGGAGCCTGCTAAAGCGCCTAGG |  |
| pSVA-△3A(75-80)-R | CAGGCTCCTCGTCTGTGTCAGAGCCAGAG |  |
| SVA-△3A(77-86)-F | GACCAAGAACCTAGGAGCGAGAACGCTTATGAC |  |
| SVA-△3A(77-86)-R | CTCCTAGGTTCTTGGTCTGTGTCAGAGCCA |  |
| Bgll II-R | GTCCAAACTTGTCTAGATTGTTAGGGAAAGAGTTGCCCG |  |
| Mlu I-F | GCTTTCTTCTGCCACGCGTGGTCTGCCGGCTCATGCTG |  |
|  |  |  |
